# Micro-compression analysis of biopolymer-producing bacteria using *Cupriavidus necator* as the model bacterium

**DOI:** 10.1016/j.tcsw.2026.100171

**Published:** 2026-02-22

**Authors:** Marketa Khyrova, Josef Sepitka, Vojtech Cerny, Jaroslav Lukes, Eva Slaninova, Tomas Plichta

**Affiliations:** a Institute of Scientific Instruments of the Czech Academy of Sciences, v. v. i., Kralovopolska 147, Brno, 612 00, Czech Republic; b Faculty of Chemistry, Brno University of Technology, Purkynova 118, Brno, 612 00, Czech Republic; c Faculty of Mechanical Engineering, Czech Technical University in Prague, Technicka 4, Prague 6, 166 07, Czech Republic

**Keywords:** Micro-compression, Surface and mechanical properties, Atomic force microscopy, Bacteria, *Cupriavidus necator*, Polyhydroxybutyrate

## Abstract

With the development of highly sensitive experimental techniques, the mechanical properties of bacterial cells have become an important research topic. However, existing models used to fit experimental data from micro-compression tests often lack accuracy. The aim of this study was to address this limitation by developing a new curve-fitting mathematical model for evaluating the mechanical properties of rod-shaped bacterial cells. The proposed model is based on a thin-shell approach and is specifically designed for the interpretation of single-cell micro-compression experiments.

To verify the applicability of the model, single-cell micro-compression tests were performed using a flat-punch nanoindenter tip larger than the bacterial cells. Atomic force microscopy (AFM) was used to obtain detailed morphological information, including precise cell dimensions required for curve fitting. As a model organism, the polyhydroxyalkanoate-producing bacterium *Cupriavidus necator* H16 was selected due to its ability to accumulate intracellular polyhydroxybutyrate (PHB) granules. For comparison, a mutant strain, *C. necator* PHB^−4^, which lacks PHB production, was also analyzed.

The results showed that *C. necator* H16 cells, with an average PHB content of 72% of dry cell weight, exhibited a Young's modulus approximately 16× higher than that of the PHB^−4^ mutant, indicating a substantial contribution of intracellular PHB granules to cell stiffness. AFM analysis further revealed that PHB-producing cells were, on average, larger in volume than the non-producing mutant. The combination of AFM and micro-compression testing enabled comprehensive characterization of bacterial cell mechanics and demonstrated a clear correlation between PHB content and mechanical behaviour.

## Highlights

• Micro-compression and AFM characterization of bacteria's mechanics and morphology.
• New mathematical model for Young's modulus (*E*) evaluation.
• Strong correlation was found between PHB content and cell elasticity.
• PHB producing bacteria exhibit 16× higher *E* than the non-producing ones.
• Optimisation of mechanical evaluation for rod-shaped cells.

## Graphical abstract

**Figure:**
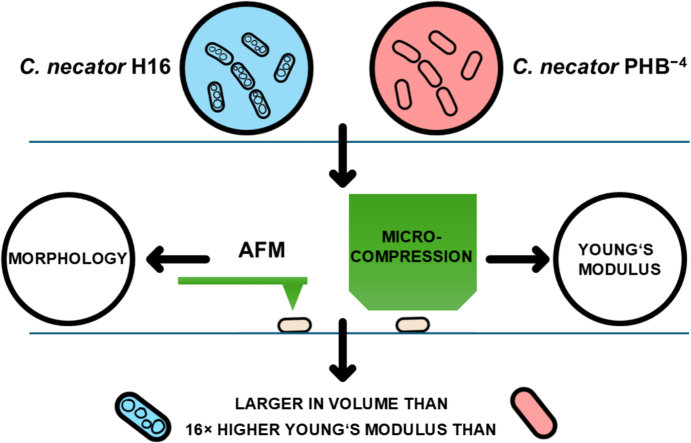

## 1 Introduction

Mechanical properties of prokaryotic cells characterize some of the key microbial processes including intracellular interactions, adhesion, biofilm formation or resilience against mechanical stress during biotechnological processes (Persat et al., 2015). These mechanical characteristics are influenced mainly by the rigid cell wall, which is composed mostly of peptidoglycan, and the outer membrane in Gram-negative bacteria. Other important influences include turgor pressure (Deng et al., 2011) or intracellular components (Rojas, 2020) like magnetosomes (Körnig et al., 2014).

To characterize the mechanical properties of bacterial cells, there have been established techniques like gel encapsulation of the bacterium (Auer et al., 2016), single bacterium cell bending caused by the flow of the liquid (Amir et al., 2014) or using osmotic shock to cause changes in the cell dimensions (Rojas et al., 2018). A few papers have also been published with the focus on the characterization of mechanical properties using a nanoindentation system to perform micro-compression tests of single cells, usually yeast or algae (Arfsten et al., 2008; Overbeck et al., 2017; Günther et al., 2016). This brings an interesting approach for the characterization of the mechanical properties based on the compression of a single cell and simultaneously controlled obtained force curve. Focusing on the micro-compression, the basic element of these tests is the use of a flat punch or flat end tip. This causes compression of the sample and not its indentation, which happens during typical nanoindentation with a non-flat tip (Uchic and Dimiduk, 2005).

Implementing this method is not difficult. However, the main problem lies in the evaluation of the obtained curves for specific shapes of the bacterium. Even though many mathematical models have been proposed describing contact mechanics, they are not applicable to some specific samples. The well-established model of contact mechanics was proposed in the late nineteen century by Heinrich Hertz (Heinrich Hertz, 1896). This model is still commonly used, even though it has many limitations in its use. It was described for the contact between two elastic spheres without the influence of adhesion and only for small deformations. On the other hand, for the elastic-plastic behaviour of materials, the Oliver and Pharr model is also frequently used (Oliver and Pharr, 1992). For biological samples, which are non-homogenous, usually viscoelastic with significant adhesion, a specific mathematical model is required to evaluate the obtained curves (Chang et al., 2016). Overbeck et al. (Overbeck et al., 2017) proposed model for the evaluation of compression of spherical cells with the linear-elastic behaviour of the cell wall, assuming the mechanical response of the whole cell.

The application of the mentioned models leads to the extraction of Young's modulus (*E*). In mechanobiology, Young's modulus is widely used as a standard quantitative parameter of cellular stiffness or elasticity, representing the ability of the cell to resist deformations under physical load (Auer and Weibel, 2017).

To be able to evaluate different shapes of the cells it is necessary to transform the mathematical model accordingly. To address this gap, we have proposed modifications to the model by Overbeck et al. (Overbeck et al., 2017) for the evaluation of mechanics of rod-shaped cells. To verify the applicability of the newly proposed approach, bacterial strain *Cupriavidus necator* was selected as a model microorganism. This bacterial strain is biotechnologically significant due to the production of secondary metabolites called polyhydroxybutyrate (PHB) in the form of intracellular inclusions (Sedlacek et al., 2019).

PHB is the first known and the most abundant example from larger group called polyhydroxyalkanoates (PHAs) (Lemoigne, 1926), which are polyesters of hydroxyalkanoic acids that may be produced by various prokaryotes as the storage of carbon and energy (Obruča et al., 2022). Additionally, these PHAs exhibit similar properties as conventional synthetic polymers but their advantage is that they are biodegradable and biocompatible, making them strategic materials for the sustainable future applications (Kucera et al., 2017).

Understanding the mechanical properties of the cells with high PHB content can provide us with important insights into how these cells function, which could potentially lead to the optimisation of conditions to increase PHB production (Bagatella et al., 2022). Among the most widely explored bacterial strains with the ability to produce PHB remains *Cupriavidus necator* (Verlinden et al., 2007). This strain is valued mainly for high and stable production of PHB with extensively explored and described production processes or various stress responses (Novackova et al., 2022a). Especially *C. necator* H16, production of PHB can reach up to 81.4% dry cell weight using, for example, orange juice as a substrate (Guzman Lagunes and Winterburn, 2016). This corresponds with the findings from 1999, which mentioned bacteria can accumulate around 80% PHB of dry cell weight (Choi and Lee, 1999). The availability of a mutant strain *C. necator* PHB^−4^ without the ability to produce PHB presents a unique opportunity. This allows the direct comparative studies under identical cultivation conditions, enabling the evaluation of mechanical changes caused by PHB accumulation (Morlino et al., 2023).

Here, we have modified the model described by Overbeck et al. (Overbeck et al., 2017) which was, as already mentioned, originally derived for spherical cells. The aim of this paper is to present the mathematical model for the accurate evaluation of the mechanical properties of rod-shaped cells. Two bacterial strains with and without PHB granules inside were used as model microorganisms. Atomic force microscopy (AFM) was used to observe the bacterial surface and obtain topographical images to determine average sizes. The relationship between PHB content and mechanical properties was analyzed, which provided a better understanding that could help with the optimisation of microbial PHB production.

## 2 Materials and methods

### 2.1 Bacterial strains and cultivation

Bacterial strain *C. necator* H16 (CCM 3726) was obtained from the Czech Collection of Microorganisms of Masaryk University in Brno, while strain *C. necator* PHB^−4^ (DSM 514) was obtained from the German Collection of the Microorganisms and Cell Cultures from Leibnitz institute. Both strains were used for the AFM and micro-compressions tests. For the bacterial inoculum, nutrient broth (NB) medium (10 g l^−1^ peptone, 10 g l^−1^ beef extract, 5 g l^−1^ NaCl) from Himedia was used. Both bacterial strains were cultivated in 100 ml Erlenmeyer flasks containing 50 ml of liquid NB media. The inoculum was cultivated for 24 h at 30 °C and 180 rpm followed by cultivation in mineral salt (MS) medium (in 250 ml Erlenmeyer flasks containing 100 ml of MS medium) for 72 h under the same conditions as the inoculum (30 °C, 180 rpm). The composition of MS is 3 g l^−1^ (NH_4_)_2_SO_4_, 1.02 g l^−1^ KH_2_PO_4_, 11.01 g l^−1^ Na_2_HPO_4_·12 H_2_O, 0.2 g l^−1^ MgSO_4_·7 H_2_O, 1 ml microelement solution (9.7 g l^−1^ FeCl_3_, 7.8 g l^−1^ CaCl_2_·2 H_2_O, 0.156 g l^−1^ CuSO_4_·5 H_2_O, 0.119 g l^−1^ CoCl_2_, 0.118 g l^−1^ NiCl_2_, 0.062 g l^−1^ CrCl_2_, 0.1 mol l ^−1^ HCl) and 1 l distilled water. Fructose solution at the concentration of 20 g l^−1^ was used as the carbon source. The MS medium, microelement solution and fructose solution were all autoclaved separately at 121 °C for 25 min.

### 2.2 Analytical procedures

The biomass concentration was determined gravimetrically from the dry weight of cells harvested from 10 ml of the bacterial culture. The bacterial culture samples were centrifuged (6000 rpm, 5 min, Hettich EBA 500), washed twice with distilled water, dried and weighed.

Additionally, the biomass growth was characterized spectrophotometrically by measuring the optical density (OD) of the bacterial suspension using NanoPhotometer P3 (IMPLEN). The absorbance of the samples was measured at 630 nm with distilled water as a blank.

Content of the PHB in the biomass was then determined using gas chromatography (Trace 1300, Thermo Scientific, column: DB-WAX 30 m by 0.25 mm) with flame–ionization detector (GC-FID) described for *C. necator* by Obruca et al. (Obruca et al., 2010).

### 2.3 Substrate preparation

Small glass Petri dishes (diameter of 40 mm) were used as substrates for cells. Firstly, they were cleaned with distilled water and ethanol. After drying, all Petri dishes were exposed to the dielectric barrier discharge (DBD) plasma for 40 s using Lifetech instrument (coil conductivity was 0.53 mS and output power 16 kW). The bottom electrode consisted of an aluminium plate, and the upper electrode, in the cylindrical shape, was made of stainless steel and designed to fit precisely into the Petri dish.

The plasma-treated Petri dishes were subsequently coated with 50 μl of 0.1 mg ml^−1^ solution of poly-l-lysine hydrobromide (PLL; Sigma-Aldrich, P6282) to cover the entire bottom surface of the Petri dish. The coated dishes were then dried in the oven at 50 °C.

### 2.4 Immobilization of bacterial cells

Bacterial cells (1 ml) were harvested and centrifuged (6000 rpm, 5 min, Hettich EBA 500), washed with phosphate buffer (PBS with composition: 8 g NaCl, 1.44 g Na_2_HPO_4_·2 H_2_O, 0.2 g KCl, 0.24 g KH_2_PO_4_ and 1 l distilled water) and centrifuged again. Bacterial pellets were then diluted in PBS according to the OD_630_ to reach the absorbance between 0.2 and 0.5. Subsequently, 1 ml of diluted bacterial suspension was placed on the PLL-treated Petri dish for 30 min. The unbound bacteria were washed off with 1 ml of PBS, after which 3 ml of PBS were pipetted into the Petri dish. The prepared sample was kept submerged in PBS for the AFM measurements. For micro-compression tests, the fluorescence probe Bodipy 439/503 was added to the bacterial suspension immediately before the fixation at final concentration of 3 μl ml^−1^. One millilitre of this bacterial suspension was quickly applied to the PLL-treated Petri dish and the subsequent steps were the same as described above for AFM measurements. However, the samples were stored in the dark.

### 2.5 Atomic force microscopy measurements

AFM was used to verify the influence of the PHB granules on the morphology, the average sizes and shape of the studied bacterial strains. All AFM measurements were performed at room temperature in PBS using NanoWizard IV (JPK/Bruker Corp.) in the quantitative imaging (QI) mode. Samples were prepared as described in the previous chapter 2.3, and the imaging took place in the Petri dish filled with PBS medium. MLCT probe (pyramidal tip A, radius 20 nm, Bruker Corp.) with the spring constant of the cantilever 0.07 N m^−1^ was used for all scans. Applied setpoint was 0.2 nN, which was gentle enough to avoid damaging the cells. All images were processed using JPK Data Processing software (JPK/Bruker corporation).

### 2.6 Micro-compression tests

For the micro-compression tests was used Hysitron BioSoft In-Situ Indenter (Bruker Corp.) in displacement-controlled mode with constant loading rate 10 μm s^−1^. Samples were prepared according to chapter 2.3. All tests were performed in prepared Petri dishes at room temperature in PBS. Nanoindenter was integrated on the inverted microscope IX73 equipped with the 120 W mercury lamp and fluorescence filter U-MWIB3 (Olympus). This microscope was used to localize the fluorescently dyed bacterial cells and determine cell sizes from the images. Tests were performed using a flat punch diamond probe with a diameter of 20 μm (Synton-MDP). It was important to secure that each bacterial cell was compressed separately without another cell under the tip.

### 2.7 Mathematical model for evaluation

Load-displacement curves were evaluated using Tribo iQ Soft Matter analysis software (Bruker Corp.). Firstly, experimental data were corrected. The starting segment of the cell compression was located and then relocated to the beginning of the coordinate system. This way the beginning point of the compression and the segment for the fitting were identified. Exact cell sizes were retrieved from the pictures taken before each compression using a fluorescent microscope. For the fitting of the experimental data Overbeck's model (Kucera et al., 2017), modified for rod-shaped bacteria, was chosen. This model is based on the model described by Lulevich et al. (Lulevich et al., 2004) for a filled capsule, which assumes a constant volume during the compression and linear elastic cell membrane deformation mostly by stretching. The equation describing dependence of the force (F) on the displacement (d) is as follows:

(1) $$F=4\mathrm{\pi E}t^{2}\varepsilon^{3}+\frac{\pi }{2\sqrt{2}}Et^{2}\sqrt{\varepsilon },$$

where E is Young's modulus, t is shell thickness and ε is relative deformation described as:

(2) $$\varepsilon =1-\frac{D_{0}-d}{D_{0}}=1-\frac{h}{D_{0}},$$

where $D_{0}$ stands for the initial diameter of the cell and h is the resulting cell height.

Overbeck, in his model for spherical cells (as thin-walled hollow spheres), assumed the greatest stress in the equatorial plane as described by Arfsten et al. (Arfsten et al., 2008) and the frictionless contact between the indenter surface, cell and substrate. As the cell wall width (t) should be small, the model simplifies the relation for inner pressure (p) and the stress at the circumferential membrane ($\sigma_{e}$) for a thin-walled hollow sphere as:

(3) $$\sigma_{e}=p-\frac{D}{4t},$$

where D is the diameter of the thin-walled hollow sphere.

To maintain the linear elastic response, the circumferential membrane stress is directly proportional to the Young's modulus (E) and to the elongation at the equator $\varepsilon_{e}$. The loading force is then given by:

(4) $$F=\frac{\pi }{4}a^{2}p,$$

where a is the diameter of the inner cylinder of the filled torus during the compression.

Since rod-shaped bacteria were examined in this study, it was assumed that the bacterial geometry is half of an ellipsoid. However, this geometry had to be converted into the shape of a filled torus to meet the condition for the calculation of the tension in the thin-walled hollow sphere. The constant volume was assumed for the transition from half ellipsoid ($V_{E0}$) to filled torus ($V_{T0}$). The initial volume of a half ellipsoid before compression was calculated according to:

(5) $$V_{E0}=\frac{1}{2}\frac{4}{3}\left(\mathrm{AB}h_{0}\pi \right),$$

where A and B are the longest and the shortest radii (length of the ellipsoid semi-axis) determined for each cell before the compression from fluorescent images, $h_{0}$ stands for the height of the cell.

**Fig. 1:**
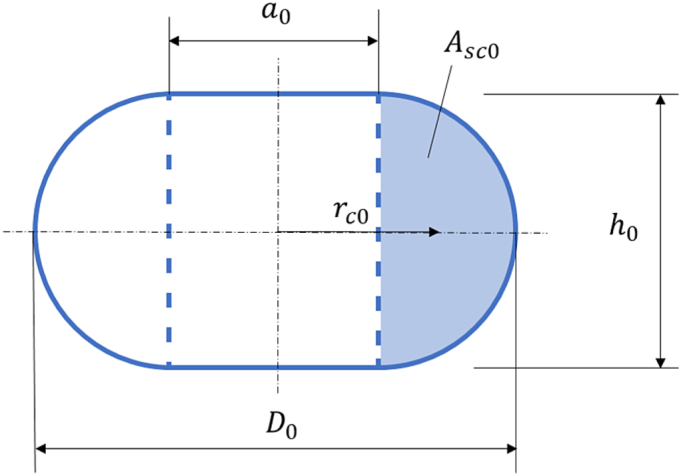
Model of bacterial cell as filled torus before compression.

Volume of the filled torus before compression is expressed as:

(6) $$V_{T0}=\frac{\pi }{4}a_{0}^{2}h_{0}+A_{\mathrm{sc}0}2\pi r_{c0},$$

where $a_{0}$ is the diameter of the cylinder inside the torus, $A_{\mathrm{sc}0}$ is the area of semicircle and $r_{c0}$ is the central radius, as illustrated by the following Fig. 1.

Before and during compression, we have again assumed no change in the volume of the filled torus ($V_{T0}=V_{T}$). Remaining variables were calculated using the formula for the equilibrium at the equatorial section of the full toroid during compression:

(7) $$p\left(\mathrm{ah}+\pi \frac{h^{2}}{4}\right)=\sigma \left(\mathrm{\pi th}+2\mathrm{at}\right).$$

**Fig. 2:**
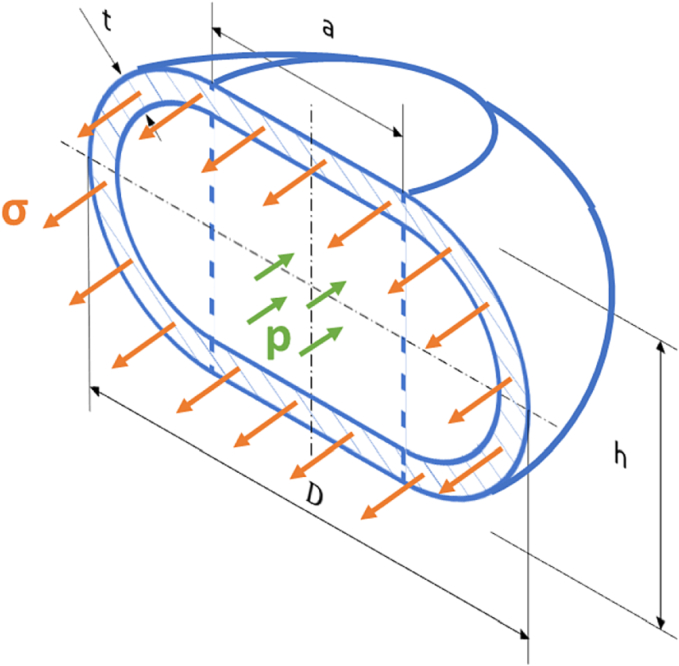
Thin-walled full torus model of a cell.

The left side of the equilibrium describes the force caused by the pressure inside the cell, where p is an internal pressure, a is the diameter of the cylinder inside the torus during compression and h is the height of the cell. The right side stands for the force acting in the thin wall of the cell, where σ is the pressure in the wall as indicated in Fig. 2.

The determination of exact *E* value was done in Python from the dependence of force (F*)* on the displacement (d), unknown Young's modulus (E) and the parameters of each cell – width of the cell wall (t), height of the cell ($h_{0}$) and the diameter of the cylinder inside the cell of a toroid shape ($a_{0}$) before the compression. Each loading curve was fitted with this model after the evaluation. Finally, all results with a lower coefficient of determination ($R^{2}$) than 90% were excluded.

## 3 Results and discussion

### 3.1 Spectrophotometric biomass density

The growth of the *C. necator* H16 and the mutant strain *C. necator* PHB^−4^ was first quantified using spectrophotometry to measure OD_630_. This provided an initial indication of culture density but served primarily as a metric prior to definitive biomass quantification by gravimetric analysis and as a critical parameter for sample dilution required for subsequent AFM and micro-compression analysis. After 72-hour cultivation, the *C. necator* H16 reached an average OD_630_ of 51.7 ± 1.1 indicating robust growth and higher biomass yield. In contrast, the mutant strain *C. necator* PHB^−4^ exhibited OD_630_ of 4.8 ± 0.5 under identical conditions. Based on OD_630_, all samples were diluted to an absorbance range of 0.2–0.5 to ensure optimal cell distribution for morphological and mechanical characterizations.

### 3.2 Determination of biomass quantity and polyhydroxybutyrate content in the biomass

**Fig. 3:**
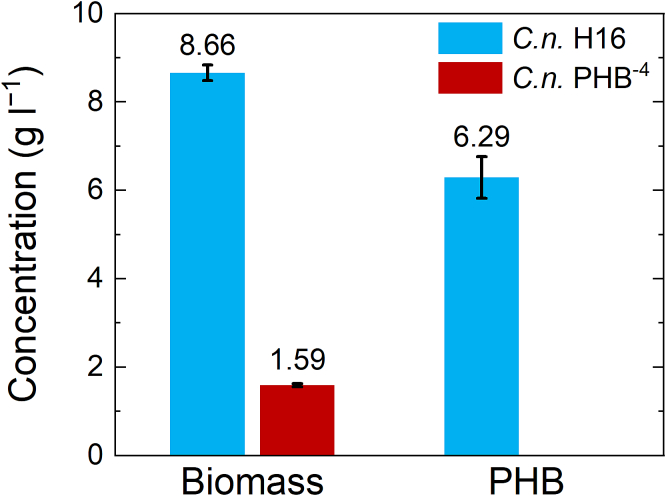
Average values of the measured dry biomass and corresponding content of PHB determined by GC. Left columns show average weight of the dried biomass, which was collected after each cultivation in the amount of 10 ml and recalculated to the g l^−1^. Right columns stand for the content of the PHB in the dried cell biomass in g l^−1^.

For further characterization of the biomass, the cell dry weight was measured gravimetrically, and the content of the PHB in the biomass was quantified using gas chromatography. These analyses followed established protocols previously described by our group (Novackova et al., 2022b; Mravec et al., 2016). The dry weight of the cells indicated that the biomass production of both bacterial strains is quite stable. However, *C. necator* H16 exhibited more than five times higher biomass production than *C. necator* PHB^−4^, with the average value 8.66 ± 0.18 g l^−1^. While mutant strain *C. necator* PHB^−4^ showed average biomass production of only 1.59 ± 0.03 g l^−1^ (Fig. 3). Content of the PHB in the biomass, determined by gas chromatography (Fig. 3), confirmed high production of PHB in *C. necator* H16, reaching 72% of the total amount of the biomass. As expected, no content of PHB was measured in *C. necator* PHB^−4^ samples.

### 3.3 Morphology of bacteria

**Fig. 4:**
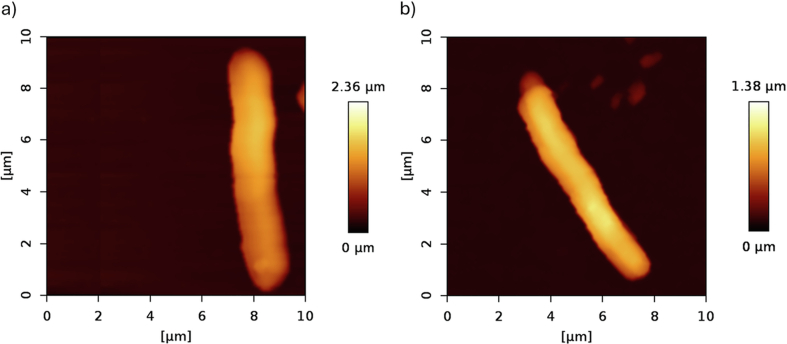
AFM images of (a) *C. necator* H16 and (b) the mutant bacteria *C. necator* PHB^−4^.

During AFM measurements, a significant influence of the PHB content on the morphology of native bacterial cells was observed. The data were collected for 12 independent cells from each strain and evaluated according to descriptive statistics but also using an independent variables *t*-test. No outliers were detected in the dataset based on Grubbs' test. Data normality was verified using Shapiro-Wilk test and homogeneity of variance was confirmed by Brown-Forsythe test (*p* > 0.05). Fig. 4 shows the differences between the two bacterial strains *C. necator* H16 and PHB^−4^. Further examples of AFM images of other *C. necator* H16 and PHB^−4^ bacteria, together with their dimensions, are shown in Supplementary material.

**Fig. 5:**
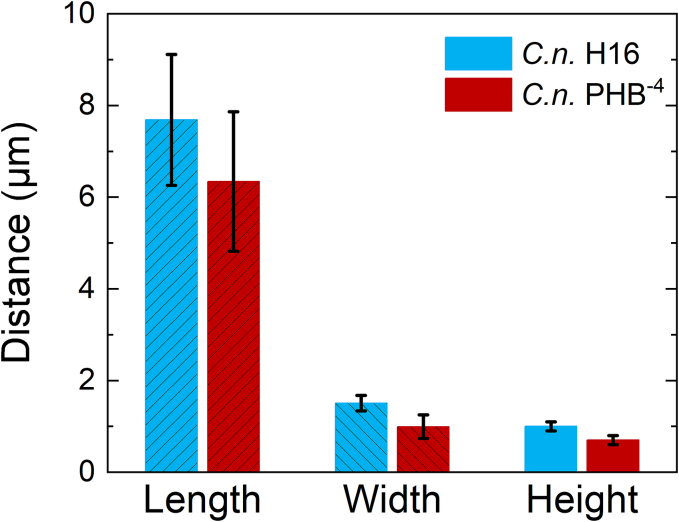
Average dimensions of each bacterial strain measured using AFM.

The average height of *C. necator* H16 was approximately 1.0 ± 0.1 μm, and its average width was 1.5 ± 0.2 μm, corresponding to a PHB content of 72% in the dry biomass. On the other hand, *C. necator* PHB^−4^, which showed no detectable PHB in the dry biomass, exhibited an average height of about 0.7 ± 0.1 μm and the average width of 1.0 ± 0.3 μm. These results suggest a significant influence of PHB granules on the morphology of the bacterial cells. In terms of cell length, both bacterial strains differed by approximately 13% on average, with the typical length of the longer *C. necator* H16 was about 7.7 ± 1.4 μm. All average dimensions are shown in Fig. 5.

**Fig. 6:**
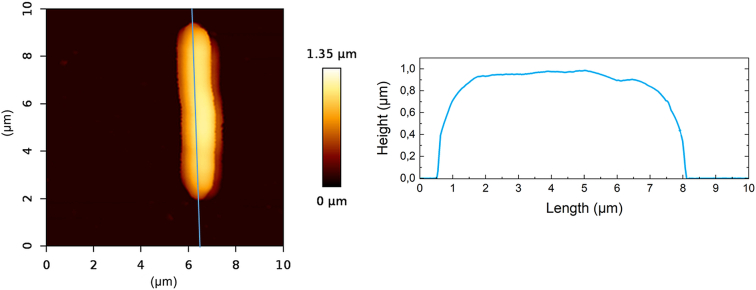
Example of *C. necator* H16 AFM image with profile on the line, where the centre 5 μm was used for the calculation of *R*_a_.

Using Gwyddion software, the average roughness ($R_{a}$) and the root-mean-square roughness ($R_{q}$) were also evaluated for both bacterial strains on the line according to Fig. 6. Both parameters were calculated from 5 μm length of the line from the middle of the bacterial cell (Nečas et al., 2020). Both surface roughness parameters ($R_{a}$ and $R_{q}$) met the criteria for application of the *t*-test. Analysis revealed a significant difference in both $R_{a}$ and $R_{q}$ between the *C. necator* H16 and the PHB^−4^ mutant strain. The average $R_{a}$ for *C. necator* H16 was determined to be 14.8 ± 4.2 nm, while *C. necator* PHB^−4^ exhibited a lower value of 6.7 ± 2.7 nm. $R_{q}$ value for *C. necator* H16 was 19.4 ± 4.9 nm, compared to 8.5 ± 3.4 nm for the mutant. These results suggest that the intracellular PHB granules influence surface roughness, contributing to the overall differences in cell morphology between the two strains.

### 3.4 Young's modulus from micro-compression tests

Data obtained from micro-compression tests were evaluated using the model described in the chapter 2.7 to determine the mechanical properties, especially Young's modulus (*E*). In this context, the Young's modulus describes cell elastic stiffness and the resistance to deformation under an applied load, where higher *E* value indicates increased mechanical rigidity and less deformable cell. The novelty of this model lies in the integration of rod-shaped cells geometry, which enhances previous approximations of spherical cells to accurately reflect the morphology of *C. necator* or other bacilli.

**Fig. 7:**
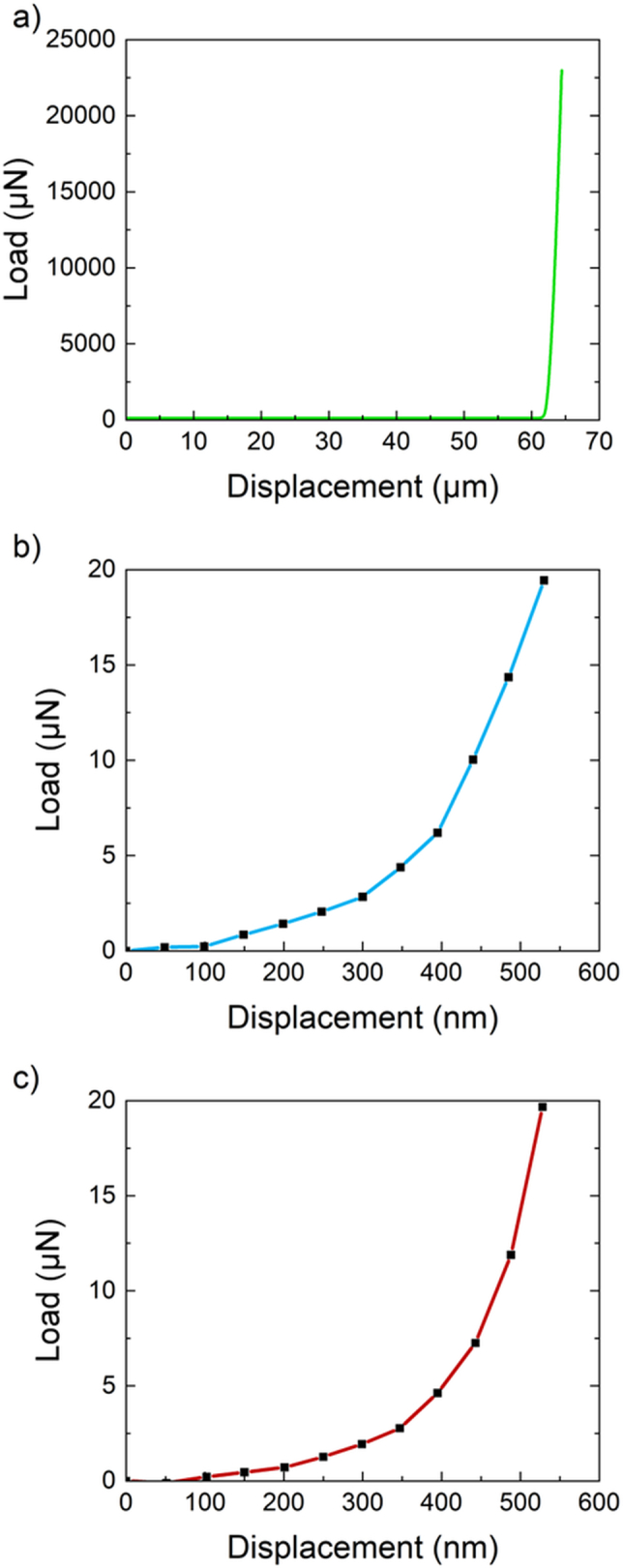
(a) The example of the whole compression curve, which had to be corrected before the fitting, (b) Load segment of the curves used for the fitting according to the mathematical model for *C. necator* H16, and (c) *C. necator* PHB^−4^.

During each compression, curves representing the dependence of the load force on the tip displacement were recorded. Data from each micro-compression test were uploaded into Python. Only the loading part of the curve was selected, as shown in Fig. 7a. The beginning of the cell compression was identified by detecting a breakpoint, where the data trend changed significantly. This point was then relocated to the origin of the coordinate system. The upper limit of loading force was set to 20 μN, since at higher values the compression of the substrate occurred. This can be observed in Fig. 7a where the load increases steeply while the displacement remains almost constant. In this way, the segment corresponding to the cell compression was identified. After these corrections, experimental data were fitted using our proposed model.

The final equation includes the height of each bacterial cell before the compression ($h_{0}$), which was measured using AFM. The averaged $h_{0}$ value for each bacterial strain was used in calculations. Exact cell dimensions (A,B) were obtained using ImageJ software from fluorescent microscope images taken prior to each compression. To determine the cell wall thickness parameter (t), transmission electron microscopy images of both bacterial strains were used. These images had been measured previously and published by Obruca et al. (Obruca et al., 2017). Using the ImageJ software, at least three values of cell wall thickness were measured from ten bacterial cells. In this way the average cell wall thickness for both *C. necator* strains was determined. The values were very similar for both strains; therefore, a single averaged value was used. The final average cell wall thickness for both bacterial strains was determined to be 23 ± 5 nm. Based on these parameters, the Young's modulus of each cell was calculated and evaluated according to the $R^{2}$ value of the data fit.

A typical segment of the loading curve selected for the fitting is shown for each bacterial strain in Fig. 7. The shape of each curve reflects the elasticity of the sample. When comparing two typical curves, the steeper slope of the loading curve can be observed for *C. necator* H16 (Fig. 7b). This indicates that this sample has a higher value of Young's modulus compared to the mutant strain (Fig. 7c).

**Fig. 8:**
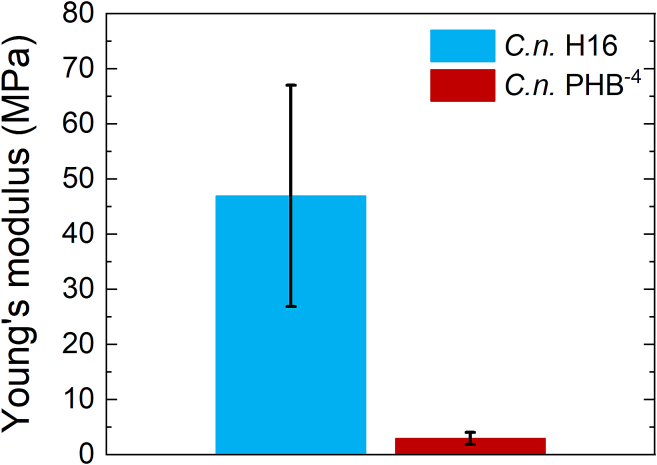
Difference between Young's modulus for both bacterial strains.

After evaluating all data obtained from micro-compression tests, the results were plotted into a graph (Fig. 8). The plotted data confirmed the initial assumption that PHB-producing bacteria exhibit higher E based on the shapes of the loading curves. The mathematical data indicated a significant difference between the two bacterial strains regarding E measured by micro-compression. The PHB-producing strain *C. necator* H16 showed average E values of 46.9 ± 20.0 MPa (measured using a 20 μm tip) with an average $R^{2}$ of 95%. This Young's modulus value is 16× higher than that of the mutant strain *C. necator* PHB^−4^, which exhibited average only 2.9 ± 1.1 MPa and the average $R^{2}$ also of 95%. The median E values were 47.3 MPa for *C. necator* H16 and 2.1 MPa for *C. necator* PHB^−4^, corresponding to 96% difference between both strains. These results may indicate that PHB granules significantly influence the overall mechanical response of the bacterial cells. The bacterial cells not only differ morphologically but also exhibit significantly different mechanical resistance. This finding is important for the biotechnological production or extraction of PHB. Higher mechanical resistance of the cells provides advantages, such as improved tolerance to shear stress generated by fluid flow in bioreactors (Lange et al., 2001). However, it should also be considered during cell disruption, as higher mechanical resistance may affect the efficiency of non-toxic PHB extraction methods.

## 4 Conclusions

This paper aimed to contribute to the mechanical characterization of bacterial cells using a micro-compression technique. In addition to a detailed description of the experimental procedure, we introduced a mathematical model for the evaluation of rod-shaped bacterial cells. The proposed modifications improve the accuracy of Young's modulus determination for rod-shaped cells and extend the applicability of the approach to a broader range of microorganisms. To validate the use of our model, we investigated PHB-producing bacterial strain *C. necator* H16 and *C. necator* PHB^−4^ as a non-producing mutant strain (both rod-shaped cells). We suggest here that the PHB-producing bacteria exhibit significantly higher values of E caused mainly due to the presence of intracellular PHB. The two studied strains not only differ morphologically, as confirmed by AFM measurements, but also exhibit significantly different values of E measured by micro-compression. Where those values differ 16× for both strains, indicating that PHB granules are responsible for this change.

This study contributes to a better understanding of how PHB granules affect the bacterial cell mechanics and provides insights that could help optimize the production and extraction of this biopolymer. The findings may support the development of more sustainable materials with reduced production costs.

## CRediT authorship contribution statement

**Marketa Khyrova:** Writing – original draft, Visualization, Validation, Methodology, Investigation, Formal analysis, Data curation, Conceptualization. **Josef Sepitka:** Methodology, Investigation, Formal analysis, Data curation, Conceptualization. **Vojtech Cerny:** Validation, Methodology, Investigation. **Jaroslav Lukes:** Writing – review & editing, Validation, Methodology, Investigation, Formal analysis. **Eva Slaninova:** Writing – review & editing, Validation, Methodology, Investigation, Conceptualization. **Tomas Plichta:** Writing – review & editing, Validation, Supervision, Methodology, Formal analysis, Conceptualization.

## Declaration of competing interest

The authors declare that they have no known competing financial interests or personal relationships that could have appeared to influence the work reported in this paper.

## Data Availability

Data will be made available on request.

## Appendix A Supplementary data

Supplementary material Image 1
