# Supplementary material for: Micro-compression analysis of biopolymer-producing bacteria using Cupriavidus necator as the model bacterium
Source: Cell Surf. 2026 Feb 22;15:100171. doi: 10.1016/j.tcsw.2026.100171 (PMC12955141; doi:10.1016/j.tcsw.2026.100171)
Supplement: Supplementary file 1 — Supplementary material [file mmc1.docx]

**Supplementary material**


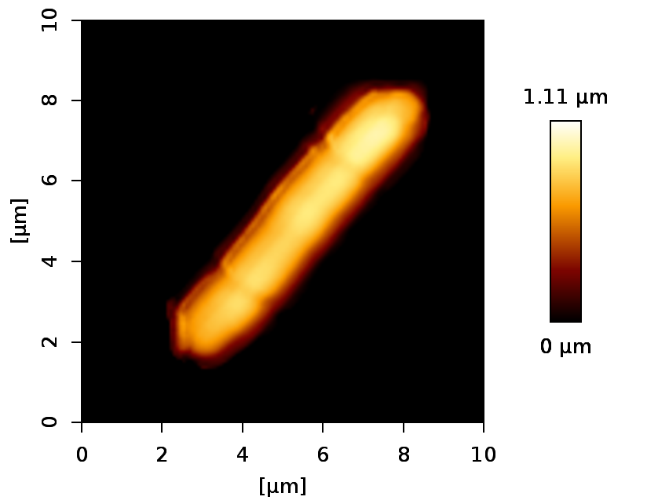


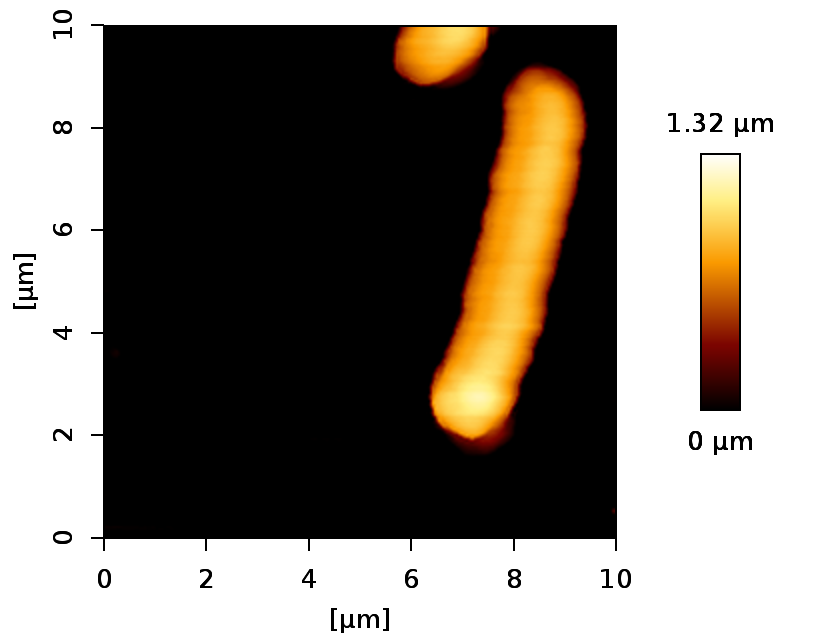


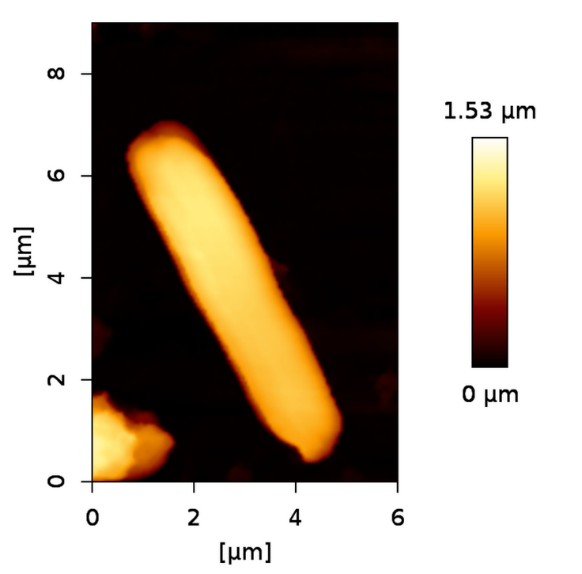


Figure S1: AFM images of *C. necator* H16 for three different bacteria (a – c).

Table S1: Dimensions of three different bacteria *C. necator* H16 (a – c) obtained from AFM images depicted in Fig. S1.

| Cell | a) | b) | c) |
| --- | --- | --- | --- |
| Length (μm) | 8.6 | 7.3 | 7.4 |
| Width (μm) | 1.8 | 1.2 | 1.6 |
| Height (μm) | 1.1 | 1.0 | 1.0 |


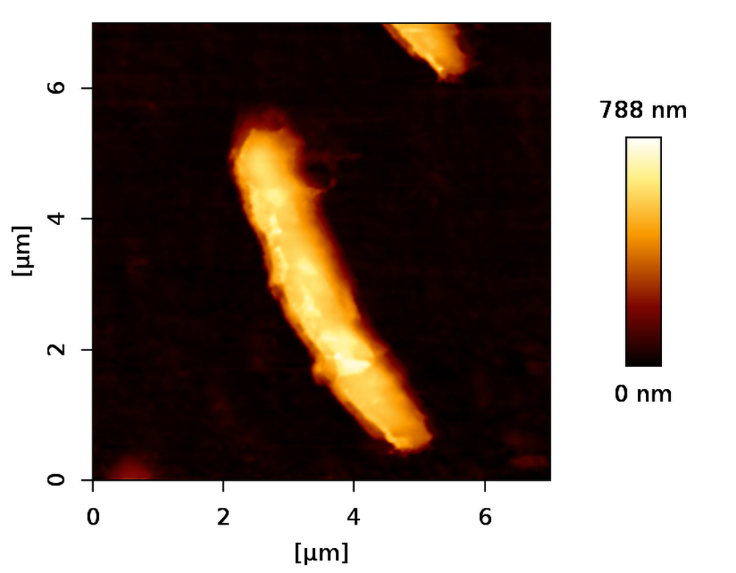


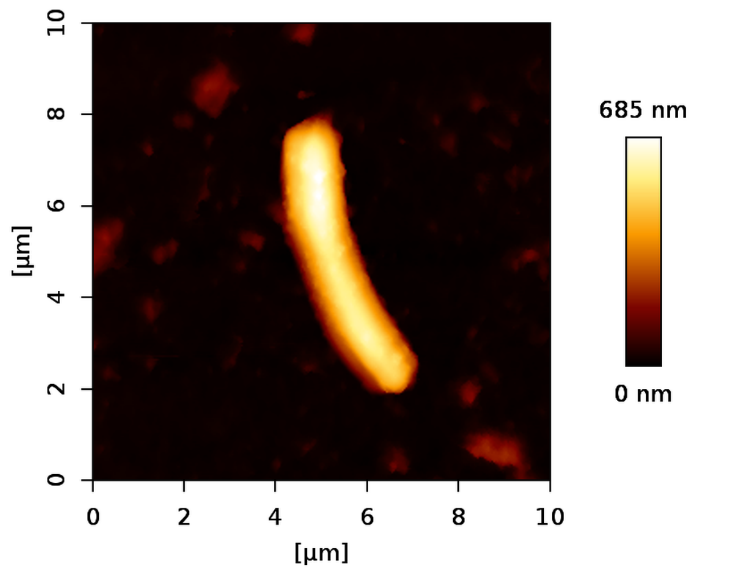


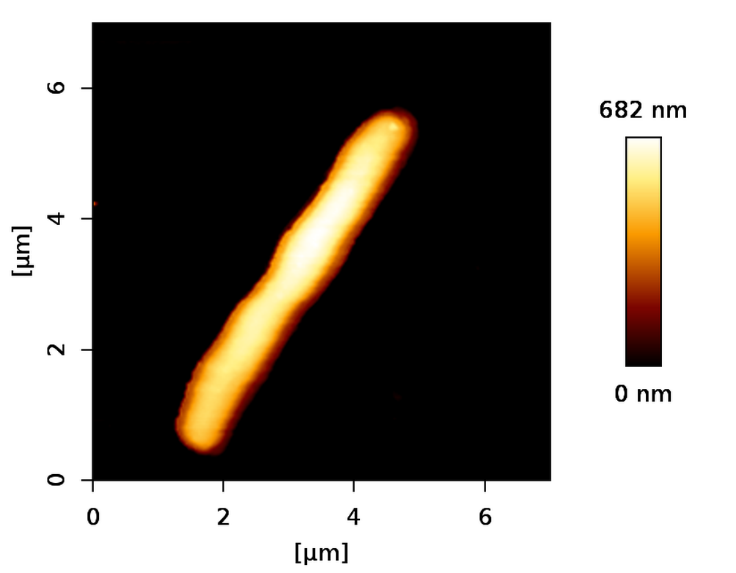


Figure S2: AFM images of *C. necator* PHB^−4^ for three different bacteria (a – c).

Table S2: Dimensions of three different bacteria *C. necator* PHB^−4^ (a – c) obtained from AFM images depicted in Fig. S2.

| Cell | a) | b) | c) |
| --- | --- | --- | --- |
| Length (μm) | 5.5 | 6.3 | 6.1 |
| Width (μm) | 1.1 | 1.1 | 1.0 |
| Height (μm) | 0.6 | 0.6 | 0.7 |
